# Condom utilization and sexual behavior of female sex workers in Northwest Ethiopia: A cross-sectional study

**DOI:** 10.11604/pamj.2015.21.50.6009

**Published:** 2015-05-25

**Authors:** Masresha Molla Tamene, Gizachew Assefa Tessema, Getahun Kebede Beyera

**Affiliations:** 1 Organization for Rehabilitation and Development in Amhara, Amhara Regional State, Bahir Dar, Ethiopia; 2 Department of Reproductive Health, Institute of Public Health, University of Gondar, Gondar, Ethiopia; 3 Department of Environmental and Occupational and Health and Safety, Institute of Public Health, University of Gondar, Gondar, Ethiopia

**Keywords:** Female sex worker, Condom utilization, AIDS, STIs, Ethiopia

## Abstract

**Introduction:**

Sexually transmitted infections are among the most important public health problems in the world. People who indulge in unsafe sex, such as female sex workers are the most at risk population groups due to multiple sexual partners and inconsistent condom use. The aim of this study was to assess condom utilization and sexual behavior of female sex workers in Gondar town, Northwest Ethiopia.

**Methods:**

A quantitative cross-sectional study triangulated with qualitative method was conducted from March 20 - April 10, 2014 in Gondar town. The quantitative data were collected through interviewing 488 female sex workers while in-depth interview was administered to collect qualitative data from 10 female sex workers. The collected data were entered into EPI-INFO version 3.5.3 and exported to SPSS version 20.0 software for analysis. Logistic regression analysis was done to determine the association between condom utilization and independent variables.

**Results:**

This study revealed that less than half (47.7%) of the respondents utilized condom with any type of client. Secondary education or above, perceiving themselves at risk of HIV/AIDS infection, having awareness that sexually transmitted infections could increase HIV infection, being tested for HIV/AIDS in the last 12 months, and having lower number of clients in a month were positively associated with condom utilization.

**Conclusion:**

This finding depicted that condom utilization was low among female sex workers. Thus, developing and implementing target oriented behavioral change and communication strategies are needed to prevent the risk of acquiring HIV/AIDS and other sexually transmitted infections in female sex workers.

## Introduction

Globally an estimated 35.3 million people are living with HIV/AIDS with 2.3 million new infections. About 69% of these populations live in sub-Saharan Africa. Striking gains have been made towards many of the 2015 targets and elimination commitments, although significant challenges remain [1, 2]. Moreover, 92% of all pregnant women living with HIV and 90% of the world's children living with HIV reside in this sub-Saharan region. In the year 2011, 71% of all AIDS related deaths worldwide were recorded in Sub-Saharan Africa [2, 3]. According to the 2011 Ethiopia Demographic and Health Survey (EDHS), the HIV prevalence in the general population is 1.5%, with urban and rural prevalence of 4.2% and rural 0.6%, respectively. The country carries one of the largest HIV disease burdens in the world [4, 5]. The Ethiopian government has identified populations who are most at-risk and/or highly vulnerable populations (MARPs) to HIV infection. MARP is defined as a group in a community with an elevated risk for HIV, often because group members engage in some form of high-risk behavior; in some cases the behaviors or HIV sero-status of their sex partner may place them at risk [6]. Within any HIV epidemic, sex workers have been one of the groups most vulnerable and at risk of HIV infection due to their multiple sexual partners spanning multiple sexual networks. High rates of other sexually transmitted infections and unsafe sexual practices further increase the probability of HIV transmission in sex workers. As a result of the risks involved and their vulnerabilities, HIV prevalence among female sex workers (FSWs) is often much higher than the general population [4, 7]. The epidemic continues to have a profound effect on female, male and transgender sex workers. FSWs are 13.5 times more likely to be living with HIV than other women. In 2008, 37% of FSWs in Amhara region were found to be HIV positive [4, 6]. Sex workers face stigma and discrimination in different forms. It is also very common for FSWs to face violence from a range of sources including clients, employers, community members, partners and other sex workers [5, 6]. Condom programming is an integral component of effective HIV prevention [1]. HIV preventive interventions targeted toward FSWs have typically focused on increasing FSWs’ condom use with commercial clients, since the contribution of commercial sex partnerships of FSWs and clients to HIV epidemics is believed to be high in many settings [8]. When used correctly and consistently, a condom is effective in preventing HIV and other STIs. Scientific evidence showed that male condom has 80% or greater protective effect against STIs including HIV [9]. However, recent surveys in several sub-Saharan Africa countries have detected a decrement in condom use and an increase in the number of sexual partners. Efforts to reduce HIV and other STI transmission related to sex work remain insufficient [2]. If FSWs do not use condoms, they will place themselves, their clients and the general population at risk of contracting HIV and other STIs since they are core transmitters in STI transmission dynamics. Despite the fact that the government has implemented different programs to educate people regarding HIV/AIDS and different preventive methods, in Ethiopia infection rate remains high, especially among MARPS. Therefore, determining condom utilization and sexual behavior of female sex workers would have strong relevance to targeted HIV prevention policies, programs, and strategies that would benefit FSWs, their clients and the general population.

## Methods

**Study design, area and period**: a quantitative cross sectional study design triangulated with qualitative in-depth interview approach was conducted from March 20 to April 10, 2014 to determine condom utilization rate and sexual behavior of female sex workers in Gondar town, Northwest Ethiopia. Gondar town is one of the tourist centers in Ethiopia where many visitors arrive throughout the year.

**Sample size and sampling techniques**: Single population proportion formula was used to compute sample size by considering the following assumptions: proportion of condom utilization among female sex workers (p) = 88% [10], 95% confidence level, 3% marginal error.

n= (Zα/2)2*P (1-P)/ d2 = (1.96)2 *(0.88)(1-0.88)/* 0.03)^2^ = 450.

Then, by adding 10% non-response rate, the final sample size (n) = 495 FSWs. For all establishments both licensed and non-licensed mapping was conducted to enumerate the number of establishments and number of FSW. We used the data which was collected by Save the Children International (SCI) and Organization for Rehabilitation and Development in Amhara region (ORDA) and HIV/ADIS Prevention and Coordination Office (HPCO) as reference. To develop sampling frame of FSWs in each establishment, we conducted our own census by involving volunteer FSWs who had experience in HaPCO, SCI, and ORDA project. After conducting enumeration and having a frame of FSWs in each establishment, we took 495 FSWs from hotels, bars, night clubs, red lights and local drinking houses proportionally. A sample of FSWs were selected purposefully for the qualitative study saturation.

**Data collection tools, procedures and quality control**: Pre-tested and structured questionnaire was administered to collect the quantitative data through interviewing FSWs. The questionnaire was first prepared in English and then translated to Amharic (local language) and back to English by different language experts to check consistency and conceptual equivalence. The Amharic version was used during the actual data collection. Twelve diploma nurses and two BSc (1 in nurse and 1 in environmental health) were involved in data collection and field supervision, respectively. Data collectors and field supervisors were trained for 3 consecutive days on data collection techniques. Five FSWs were recruited as guider to reach sampled FSWs. The collected data were checked for completeness and relevance on daily basis by field supervisors. In addition, in-depth interviews were conducted on a total of ten female sex workers until saturation had been reached using semi-structured interview guide. The interviews were conducted in places where there were no interruptions, and tape recorded.

**Data processing and analysis**: Data were coded and entered into Epi-info version 3.5.3 and exported to and analyzed using SPSS version 20.0 software package. Both bivariate and multivariate logistic regression analysis were done to determine the effect of various independent variables on condom utilization. The results were presented in the form of tables, figures and text using frequencies and summary statistics such as mean, median, standard deviation and percentage to describe the study population in relation to relevant variables. The degree of association between dependent and independent variables were assessed using odds ratio with 95% confidence interval and p-value 0.05. For the qualitative part, data collected through in-depth interviews were transcribed and cleaned manually after being recorded into the computer. Then, these data were coded and categorized using open code software version 3.4. Finally, the data were sorted and descriptive analysis was carried out to find the core meanings. The findings were interpreted using thematic analysis.

**Ethical consideration**: Ethical clearance was obtained from the Research and Ethical Review Committee of the Institute of Public Health, University of Gondar. Formal letters of cooperation were also obtained from Gondar Town Administration Mayor Office, Health office, Women, Children, Youth and Social Affairs Office. Informed verbal consent was obtained from each FSW after giving clear explanations about the objective and importance of the study. Confidentiality was maintained by using codes instead of any personal identifiers.

## Results

**Socio demographic and economic characteristics of female sex workers**: Out of 495 selected FSW, 488 were involved in the study (response rate = 98%). The mean age of the respondent was 25.63 (SD = 5.6) of which nearly one third of them were found in the age range of 20-24 years. Concerning their educational status, 115(23.6%) were unable to read and write (Table 1).

**Table 1:** Socio demographic characteristic of FSW in Gondar Northwest Ethiopia, 2014

Variables	Frequency	percent
**Age group**		
15-19	59	12.9
20-24	164	33.6
25-29	164	33.6
30-34	58	11.9
≥ 35	43	8.8
**Type of working establishments**		
Hotel	107	21.9
Bar	138	28.3
Night club	22	4.5
Red light	77	15.8
Local drinking house	144	29.5
Place of growth		
Rural	247	50.6
Urban	241	49.4
Educational status		
Unable to read & write	115	23.6
Able to read and write	116	23.8
Primary education	139	28.5
Secondary or above	118	24.2
**Marital status**		
Single	274	56.1
Married	27	5.5
Divorced	137	28.1
Widowed	34	7.0
Separated	16	3.3
Given birth		
Yes	202	41.4
No	286	58.6
**Family size of FSW**		
≤5	476	97.5
>5	12	2.5
**Family occupation before engaged in sex work**		
Farmer	229	46.9
Daily Laborer	95	19.5
Civil servant	55	11.3
Merchant	74	15.2
Driver	20	4.1
Others	15	3.1
**Monthly income of FSW(ETB)**		
≤1500	140	28.7
1501-2500	110	22.5
2501-4000	127	26
≥4001	111	22.7

**Knowledge about HIV/AIDs and STI transmission and prevention methods**: One hundred eight three (37.5%) of the respondents had knowledge about HIV and STI transmission and prevention methods. Also, 170(34.8%) of them knew at least the three major signs and symptoms of STI. About 70% of the respondents reported that STI increases HIV infection. A 21 year old FSW said “I was visiting the clinic for STI treatment. STI and HIV are similar in mode of transmission and STI will be treated if we are infected with it”. Both HIV and STI transmission methods are unsafe sex, having multiple sexual partners and the prevention methods are abstain, faithful and proper condom utilization and avoiding using sharp objects in common". Another 21 years old FSW said “HIV can be transmitted through unsafe sex. However, STI transmission is not the same as HIV; it is transmitted through seating on hot stone or place and urinating towards the moon and its treatment is traditional than modern”. All the respondents had heard about the condom before; Friends 77.5%, health professionals 76% and mass media 74.2% were among the top sources of information. Though 64 and 25 FSWs practiced anal and oral sex respectively with their partners, only 34 and 3 of them used condoms, respectively. Among those who used condoms, 99.2% FSWs used it for HIV/AIDS prevention, while 80.1% used it for the prevention of other STIs. Nearly 44% of the respondents perceived that they were at risk of HIV infection (Table 2). An 18 years old FSW said that “ߪߪdoing as sex worker by itself is at risk of getting HIV infection due to multiple sexual partner; there may be condom breakage and slipping during sex and also we may have unsafe sex by the influence of different factors.” In contrary, 24 years old FSW said “ߪߪߪ I am not at risk of getting HIV/AIDS because I use condom properly with any of my sex clients throughout this work. So, I think that I am safe from HIV/AIDS; the main thing is using condom properly.”

**Table 2:** Knowledge of FSWs about HIV/AIDS and STI transmission and prevention methods, Gondar town, Northwest Ethiopia, 2014

Variable	Frequency	Percent
**Had knowledge about HIV and STI transmission and prevention methods**		
Yes	183	37.5
No	305	62.5
**Knowledge of at least three STI sign and symptoms**		
Yes	170	34.8
No	318	65.2
**Other STIs increase HIV infection**		
Yes	341	69.9
No	147	30.1
**Source of information about condom**		
Health professional	371	76.0
From friend	378	77.5
Client	316	64.8
Health institution	334	68.8
Mass media	362	74.2
Family	77	15.8
Others	10	2.0
**Type of sex practiced**		
Vaginal	488	100.0
Anal	64	13.1
Oral	25	5.1
**Reason for using condom**		
HIV prevention	484	99.2
STI prevention	391	80.1
To avoid unwanted pregnancy	423	86.7
**Perceive that they are at risk of HIV**		
Yes	213	43.6
No	275	56.4

**Service Utilization**: Majority (85%) of FSWs had ever used any type of reproductive health services, and 381(78.5%) were tested for HIV in the last 12 months. “……… I have used reproductive health services like HIV testing and family planning counseling; also I got condom from health institutions and I can access information about HIV/AIDS ”.. The reason I undergone HIV test is to know my status and it encourages me to use condom (25 years FSW).“ “….I have parted in peer education session which was led by our friend and I got a lot of things on it about condom utilization, benefits of testing HIV and STI, and how to negotiate with clients and about methods of transmission and prevention of STI(21 years old FSW).” Majority (91.8%) of the respondents recalled shops as places where there was availability of condoms (Table 3).

**Table 3:** Health service utilization by FSWs in Gondar town, Northwest Ethiopia, 2014

Variables	Frequency	Percent
**Use reproductive health services**		
Yes	415	85.0
No	73	15.0
**Had access to get condom**		
Yes	457	93.6
No	31	6.4
**Know places where to get condom**		
Yes	467	95.7
No	21	4.3
**Can access condom at working establishment**		
Yes	421	86.3
No	67	13.7
**Place where condoms were available**		
Shop	448	91.8
Pharmacy	334	68.4
Health institution	342	70.1
Hotel	316	64.8
Friend	261	53.5
health professional	195	40.0
**Tested for HIV in the last 12 month**		
Yes	381	78.1
No	107	21.9
**Reasons why underwent HIV test (n = 381)**		
To know my status	305	62.5
Due to Illness	71	14.5
For pregnancy	25	5.1

**Condom utilization and sexual behavior**: Of the total FSWs who had sex in the past one month, 411(84.2%), 351(71.9%), and 160(32.8%) utilized condom with their non-regular partners, regular partners, boyfriends/husbands, respectively. The overall consistent and correct condom utilization among FSWs was 233(47.7%). “……I ask every client to use condom. If he accepts we use it. If he does not, I cannot force him. Whether to use condom or not depends on the client.” (FSW aged 30 years). On the other hand, 19 year old FSW “I used condom all the time with any clients. If a boy asked me to have sex without condom I consider he is HIV/AIDS positive and I would gave attention in any sexual action.” The number of a FSW's clients range from 3 to 70, with median of 13 clients per month. Within the last one month, 32% and 22.3% of FSWs faced incidence of condom breakage and slipping during sexual intercourse, respectively. In this study, 360(73.8%) of the respondents had drunk alcohol in the last month of which 119(33.1) had drunk on daily basis. Violence was common among FSWs. In this study, 120(64.8%) of FSWs banned payment after the intercourse, and 104(55.6%) sustained physical violence (Table 4). A 21 year old FSW Said “…I chew that to drink a lot, to avoid sleeping and to easily say ok my sex clients…. I would be intoxicated when I drank a lot though I tried to avoid drinking much. Any how it is difficult; I would be intoxicated unintentionally; after that I couldn't know what is going to happen. “.. We are also forced by establishment owner to drink much….” Sexual intercourse other than vaginal was reported by the study participants. “I had been asked by my clients to suck his penis and to have anal sex. I underwent anal sex with condom but I refused to practice oral sex with him….” 19 years old FSW. Another FSW said “I consider that anal and oral sex could not transmit HIV. So, I am inconsistent with condom use.”

**Table 4:** Condom utilization and Sexual behavior of FSWs in Gondar town, Northwest Ethiopia, 2014

Variable	Frequency	Percent
**Median number of clients in the last one month**		
≤13	306	62.7
>13	182	37.3
**Used condom for sex with non-regular partners in the last one month**		
Yes	411	84.2
No	77	15.8
**Used condom for sex with regular partners in the last one month**		
Yes	351	71.9
No	137	28.1
**Used condom for sex with boyfriend /husband in the last one month**		
Yes	160	39.0
No	247	50.6
I don't have boyfriend/husband	81	16.6
**Used condom for sex with all types of partners**		
Yes	233	47.7
No	255	52.3
**Faced incidence of condom breakage during sex in the last one month**		
Yes	160	32.8
No	328	67.2
**Faced incidence of condom slipping during sex in the last one month**		
Yes	109	22.3
No	379	77.7
**Amount of payment for one night(ETB)**		
≤ mean (203)	322	66
> 203	166	34
Common clients		
Driver	400	82.0
Merchant	375	76.8
Daily laborer	275	56.4
Civil servant	237	48.6
Student Others	182 16	37.3 3.3
**Drank alcohol in the last one month**		
Yes	360	73.8
No	128	26.2
**Faced violence in the last one month**		
Yes	185	37.9
No	303	62.1
**Type of violence**		
Physical	104	55.6
Sexual & refusal to pay money	120	64.8
Psychological	90	48.1

**Factors associated with condom utilization among female sex workers**: In the bivariate logistic regression analysis age; place of growth, marital status, having additional job, educational status, years worked as FSW, monthly income, knowledge about HIV/AIDS and STI transmission and prevention methods, knowledge about sign and symptoms of STIs, having awareness that STI will increase HIV infection, perception of at risk of HIV infection, Having tested HIV/AIDS in the last 12 months, number of sex partners in the month were significant at p-value < = 0.2. However, in the multiple logistic regression, only educational status, knowledge on HIV and STI transmission and prevention, HIV risk perception, awareness of STI will increase HIV infection, HIV testing, and number of client remained as a statistically significant factor for condom utilization. Respondents who had secondary educational status or above had 3.7 times higher odds of using condom than those who were not able to read and write (AOR:3.7, 95%CI (1.69, 8.25)). Having good knowledge of HIV/AIDS and STI transmission and prevention were associated with condom utilization; respondents who have good knowledge of HIV/AIDS and STI transmission and prevention had about 2 times higher odds of using condom than who did not. (AOR: 1.9, 95%CI (1.04, 3.32)). Those FSWs who reported STI can increase HIV infection had about 3 times higher odds of using condom than those who didn't (AOR:3.11, 95%CI (1.63, 5.94)). Those FSWs who did not perceive themselves at risk of getting HIV/AIDS had about 6 times higher odds of using condom than those who perceive themselves at risk of HIV infection (AOR:5.8, 95%CI (3.18,10.53)). FSWs who participated in HIV/AIDS prevention program had about 13 times higher odds of using condom than those who did not (AOR:13.3,95%CI(7.33,24.10)). Those FSWs who were tested for HIV/AIDS in the last 12 months had 3.4 times higher odds of using condom than who were not tested (AOR:3.4, 95%CI(1.51,7.78)). FSWs who had less clients in a month than the median were 2.5 times the odds of using condom than those who had more clients than median (AOR:2.5, 95% CI (1.42,4.25)) (Table 5).

**Table 5:** Factors affecting condom utilization among FSW in Gondar town Northwest Ethiopia, 2014

Variables	Condom Use	COR(95% CI)		AOR( 95%CI)
	Yes	No		
**Place of birth**				
Rural	99	148	1	
Urban	134	107	1.87(1.31, 2.68)	
**Marital status**				
Single	149	125	1	
Married	13	14	0.78(0.35,1.72)	
Divorced	51	86	0.45(0.33,0.76)	
Widowed	15	19	0.67(0.32,1.36)	
Separated	5	11	0.38(0.13,1.13)	
**Educational status**				
Unable to read &write	28	87	1	1
Able to read &write	32	84	1.18(0.66,2.13)	0.95(0.42,1,17)
Primary education	86	53	5.04(2.92,8.70)	2.17(1.02,4.57)*
Secondary & above	87	31	8.72(4.83,15.75)	3.74(1.68,8.25)*
**Working year as FSW**				
< 1 year	23	22	1.32(0.68,2.57)	
1 year	41	31	1.67(0.95,2.94)	
2 year	55	75	0.93(0.58,1.49)	
3 year	46	41	1.42(0.84,2.41)	
4 or above year	68	86	1	
**Monthly income (ETB)**				
<= 1500	49	91	1	
1501-2500	53	57	1.73(1.04,2.88)	
2501-4000	66	61	2.01(1.23,3.29)	
> = 4001	65	46	2.62(1.57,4.38)	
**HIV &STI transmission &prevention methodknowledge computed variable**				
Yes	127	56	4.26(2.87,6.30)	1.86(1.04,3.33)*
No	106	199	1	1
**Knowledge of three major sign & symptom of STI**				
Yes	114	119	3.40(2.30,5.04)	
No	56	199	1	
**Awareness STI will increase HIV infection rate**				
Yes	205	136	6.41(4.02,10.20)	3.11(1.63,5.94)*
No	28	119	1	1
**Perceiving at risk of getting HIV**				
Yes	72	141	1	1
No	161	114	2.77(1.91, 4.01)	5.78(3.18,10.51)*
**Tested HIV in the last 12 months**				
Yes	219	162	8.98(4.94,16.32)	3.43(1.51,7.78)*
No	14	93	1	1
**Number of sex partners per month**				
< = 13	148	99	2.74(1.90,3.96)	2.46(1.42,4.25)*
>13	85	156	1	1
**Experienced Violence**				
Yes	75	112	1	
No	158	143	1.65(1.14,2.39)	

## Discussion

This study aimed to assess condom utilization and sexual behavior of female sex workers in Gondar town. The result showed that 47.7% (CI 42.8%, 52.4%) of the respondents utilized condom with any type of clients This result is similar with a study conducted in Ghana 49.6% [11], North Mexico (43%) [12] and South Africa (43%) [13]. However, this finding is lower than studies conducted in South Asia (86.9%) [14], South Indian (81.7%) [15], Hubei-China (74.9%) [16] and Bangladesh (58.9%) [17]. The possible reasons for this different might be linked to difference in socio-demographic and economic characteristics. Similar with other studies [11, 14, 17], trust of client, client objection, seeking better satisfaction and substance use were amongst the major reasons acknowledged by FSWs for their non consistent condom use. Educated SFWs had higher odds of using condom, which might be due to the fact that education may provide confidence to use condoms with clients. In addition, education might increase ability to condom negotiation. This finding is in line with studies done in Mexico [12], South Asia [14], Ghana [11], South Africa [13] and Central Ethiopia [8]. Among the determinants explored, knowledge about HIV/AIDS and STI transmission and prevention methods were identified as factors determining whether to use or not to use condoms consistently. The respondents reported that knowledge about the transmission and prevention methods of HIV/AIDS and STI affects significantly the utilization of condom. Therefore this study revealed that having knowledge about the transmission and prevention of both HIV and STI have positive effect on utilization of condom. That means if they had knowledge of both they try to use condom consistently and correctly. This result was supported by a study conducted in Bangladesh [17] and Ghana [11]. Possible reasons would be that the knowledge would increase utilization of condom correctly and consistently.

This study identifies that awareness of the fact that STI will increase the probability of HIV acquisition was positively associated with condom utilization. A similar was obtained in a study conducted in Andhra Pradesh [18]. This might be explained by awareness of precursors for HIV acquisition will encourage them to use condom consistently. This study revealed that FSW utilization of health care system especially being tested for HIV in the last 12 months significantly associated with condom utilization. Those who had got HIV/AIDS testing were more likely to use condom than who were not tested. This could be as the result of the counseling they got in health institutions and the motivation to reduce risk by using condom consistently and correctly. This is similar a study in Cambodia-South Asia [14]. Numbers of clients were the determinant factors of condom utilization, in this study we used the median as a cut of point as most studies used to know the effect of client number to condom utilization. Those FSWs who had less number of clients had more odds of using condom than who had more clients. This finding is supported with the studies conducted at Cambodia [14], India [18], and Central Ethiopia [8]. This might be as the number of client increases their negotiation skill will be decreased due to tiredness and to satisfy their clients’ need. They may consider themselves at high risk of infection due to multiple sex partners. FSWs reported that their common clients were; driver merchant, daily laborer and civil servants were the commonest client who visited FSW. This is similar with other studies in our country.

## Conclusion

This finding depicted that female sex workers condom utilization was low with any type of client whether non regular, regular or boy-friend/husband, which places not only FSW but also the general population at high risk of HIV/AIDS and STIs. Moreover, Primary and secondary or above education, knowledge about HIV and STI prevention and transmission methods, Perceiving at risk of HIV infection, awareness of STI will increase HIV infection, participating in any HIV prevention program, being tested for HIV, and having lower number of clients were positively associated with condom utilization.
